# Do slower movers have lower reproductive success and higher mutation load?

**DOI:** 10.1002/evl3.87

**Published:** 2018-11-12

**Authors:** Carly B. Walsh, Katrina McGuigan

**Affiliations:** ^1^ School of Biological Sciences The University of Queensland Brisbane 4072 Australia

**Keywords:** *Danio rerio*, ENU mutagenesis, *U*_crit_, viability selection, zebrafish

## Abstract

Deleterious mutations occur frequently in eukaryotes, resulting in individuals carrying multiple alleles that decrease their fitness. At a population level, if unchecked, accumulation of this mutation load can ultimately lead to extinction. How selection counters the accumulation of mutation load, limiting declines in population fitness, is not well understood. Here, we use manipulative experiments in zebrafish (*Danio rerio*) to investigate the opportunities for selection on mutation load. Inducing high mutation load through mutagenesis, we applied one generation of within‐family selection on locomotor performance and characterized both the direct response to this selection and the indirect response of reproductive success. Offspring of slow swimming parents exhibited age‐dependent declines in swimming speed, whereas their cousins, with faster swimming parents, did not. This pattern mimics previously documented differences between high and low mutation load populations of zebrafish, suggesting that slow swimming siblings inherited (and transmitted) more mutations than their faster swimming siblings. Crosses among offspring of slow swimming fish had, on average, <75% of the reproductive success of crosses among offspring of fast swimming parents, or crosses of offspring of slow swimmers with offspring of fast swimmers. This evidence of mutationally correlated swimming speed and reproductive success reveals the potential for concordant selection on mutation load through different fitness components. There was no evidence that crosses within families (where parents potentially shared the same mutations inherited from their common ancestor) had lower reproductive success than crosses among families, suggesting that viability selection was not acting predominantly through lethal recessive homozygotes. Rather, patterns of reproductive success are suggestive of effects of mutation number per se on embryo viability. Overall, our results highlight the potential for early life mortality to remove deleterious mutations, and the need to account for this mortality when investigating the evolutionary dynamics of mutation load.

**Impact Summary**

It is well known that harmful mutations occur frequently in animals and plants, with individuals carrying multiple harmful mutations inherited from their parents. If unchecked, the accumulation through time of this mutation load results in population extinction. Although we know that such mutational meltdown is prevented in most populations, we have a poor understanding of how, as selective deaths of many individuals might result in reproduction too low for population persistence. We introduced high mutation load into a population of zebrafish to investigate how selection acts to remove this load. Through a series of experiments involving three generations of fish, we inferred that individuals expected to have inherited relatively many mutations were less likely to survive to hatching than individuals inheriting relatively few mutations. Although early mortality is common in many plant and animal taxa, there is little information on the contribution of mutation load to this mortality. Early‐life mortality might be a very effective way of removing mutations because these individuals have relatively little influence on the life‐long resource access of survivors. Our experiments also provided evidence that mutation load affects swimming speed in zebrafish, although indirectly through effects on speed‐at‐age relationships. The evidence that mutation load impacts on swimming speed is an important validation of the expectation that whole‐organism performance traits, like locomotion, are important indicators of mutation load. Finally, our results contribute to an emerging story that mutations might contribute to the phenotypic variation we observe in natural populations through impacts on timings across the lifecycle, including delayed mutation or accelerated aging.

A variety of experimental approaches have consistently revealed that mutations arise frequently, and that they typically decrease fitness (Eyre‐Walker and Keightley 2007; Halligan and Keightley 2009). This pervasive presence of deleterious mutation suggests that a major role of selection is to limit fitness decay. Indeed, when natural selective processes are disrupted, populations typically decline in fitness (Halligan and Keightley 2009; McGuigan and Blows 2013; Bosshard et al. 2017) and show elevated frequencies of putatively deleterious alleles (Marsden et al. 2016; Makino et al. 2018). Deleterious mutation is thought to play an important role in phenomena ranging from the evolution of sex and sexual selection through population persistence (Rowe and Houle 1996; Schultz and Lynch 1997; Whitlock 2000; Agrawal 2001; Whitlock and Agrawal 2009). Despite this, the microevolutionary processes acting on mutations, and the demographic and ecological consequences of these processes, remain poorly understood (Agrawal and Whitlock 2012).

The efficacy of selection on mutation load and the direct impacts of this selection on population growth depend on how many mutations are removed by each selective death (i.e., per individual that contributes no offspring to the next generation), and whether individuals that die a selective death reduce fitness of conspecifics through consumption of limiting resources (Agrawal and Whitlock 2012). Sexual selection on males might be an important mechanism of mutation load reduction because males that fail to reproduce do not limit access of other males to the necessary resource—females. Empirical studies, on both individual mutations and genome‐wide mutation load, have provided conflicting evidence that male sexual selection does (e.g., Whitlock and Bourguet 2000; Hollis et al. 2009; McGuigan et al. 2011a; Almbro and Simmons 2014; Lumley et al. 2015; Grieshop et al. 2016; Dugand et al. 2018), or does not (e.g., Radwan et al. 2004; Arbuthnott and Rundle 2012; Chenoweth et al. 2015) reduce mutation load, where contrasting results might actually be explained by the population's load of low fitness alleles (Long et al. 2012).

More generally, the ecological and evolutionary consequences of the interplay between resource competition and selection on mutation load (Agrawal and Whitlock 2012) suggest that selection on condition‐dependent phenotypes could be important for reducing mutation load. Condition, the total pool of resources available for allocation to any fitness‐enhancing phenotype, is determined by many processes and will therefore be affected by relatively many mutations (Rowe and Houle 1996; Tomkins et al. 2004). Many phenotypes, including male sexual displays, life history traits, and whole‐organism performances, are condition dependent (Houle 1991; Rowe and Houle 1996; Hunt et al. 2004; Tomkins et al. 2004; Whitlock and Agrawal 2009; Husak and Lailvaux 2017; Lailvaux and Husak 2017), suggesting the potential for concordant selection on mutations affecting condition to increase the efficacy of selection.

In contrast to sexually selected and life history traits, some condition‐dependent performance traits, including locomotor performances, are expressed throughout life, allowing selection to act earlier in life to limit resource depletion by high mutation load individuals. Locomotor performance is hypothesized to directly impact on individual fitness, with some empirical support for this (Husak and Fox 2008; Irschick et al. 2008). Morphological, physiological, and behavioral traits are predicted to come under indirect selection through their influence on performance (Arnold 2003; Walker 2007), an expectation consistent with performance having a highly polygenic basis, and potentially capturing a broad range of deleterious mutation. Two studies of *Drosophila* have considered the effect of naturally accumulating mutations on locomotor activity, with no evidence that mutation decreased activity (Huey et al. 2003; Latimer et al. 2014). A previous study in zebrafish using a measure of locomotor performance expected to reflect physiological capacity suggested that mutation load might indirectly affect maximum locomotor performance through changes in developmental timing across the lifecycle, but evidence of deleterious fitness consequences of such changes were lacking (McGuigan and Aw 2017).

Selection operating on gametes or early in development is another mechanism predicted to be effective at removing mutation load without negatively affecting population growth, as load is removed with little impact on resource availability for survivors (Agrawal and Whitlock 2012). Phenotypic differences exist between individuals that die before maturity and survivors (Diaz et al. 2003; Mojica and Kelly 2010; Polak and Tomkins 2013). Although the role of genetic causes of viability have received attention from the perspectives of inbreeding avoidance (e.g., Fitzpatrick and Evans 2014; Firman and Simmons 2015), speciation (e.g., Corbett‐Detig et al. 2013; Christie and Strauss 2018; Pulido‐Santacruz et al. 2018), and selection on immune loci (e.g., Chae et al. 2014; Lukasch et al. 2017), the role of gamete or early life viability in reducing mutation load has received limited attention (Agrawal and Whitlock 2012; Plough et al. 2016; Alavioon et al. 2017; Immler and Otto 2018). Direct observation of such early acting selection will be impossible in many taxa, while characterizing the genetic basis of viability is challenging given that fitness is highly polygenic, deleterious alleles are individually rare, and may have small effects (Visscher et al. 2010; Csillery et al. 2018).

In this article, we investigate the potential for selection on adult swimming speed, and on early life survival to contribute to reducing mutation load. We used mutagenesis to generate populations of zebrafish, *Danio rerio*, with multiple, putatively deleterious mutations, each at high frequency (∼50%) within the local population. By increasing the frequency of individual alleles, mutagenesis (or mutation accumulation) increases the power to detect phenotypic (fitness) effects of mutation load (e.g., Radwan 2004; Sharp and Agrawal 2008; McGuigan and Blows 2013). We used bidirectional artificial selection to separate siblings into high versus low swimming performance groups. If mutation load affects performance, we expected poorer performing individuals to have inherited a higher mutation load than their better performing siblings. We, therefore, determined whether selection on swimming performance resulted in correlated responses in reproductive successes, predicting that offspring of slow‐swimming parents would have lower reproductive success. This experiment allowed us to consider whether mutations had concordant effects on different fitness components, and whether selection on adult speed and embryo survival could act to reduce mutation load.

## Methods

### POPULATION HISTORY AND APPLICATION OF SELECTION

The mutagenesis treatment has been described elsewhere (McGuigan and Aw 2017). Briefly, WIK strain (Rauch et al. 1997; Trevarrow and Robison 2004) males were exposed to 3 mM ENU for 1 h, then allowed to recover for one month (to ensure offspring inherited only germline mutations: Solnica‐Krezel et al. 1994) before being bred with an unrelated, not mutagenized, female from the same WIK population. Each full‐sib family was reared in a single 3.5L Techniplast S.p.A (Buguggiate, Italy) tank on a recirculating water system, at a density of ∼10 fish per liter, at 28°C, with fish fed three times a day on a juvenile then adult diet (for diet details, see Conradsen et al. 2016). Families derived from each ENU mutagenized male were treated as independent replicates of the divergent selection treatments described below. There was little among‐family variance in swimming speed or body size in the WIK population prior to ENU treatment, but variance increased markedly following mutagenesis (McGuigan and Aw 2017). Therefore, selection was expected to be acting predominantly on novel mutational variance.

Critical swimming speed, *U*_crit_ (Brett 1964), was determined for a total of 201 fish from eight ENU families when fish were 103–148 days postfertilization (dpf; median 127 dpf). *U*_crit_ was assessed using a stepped velocity test in a Loligo Systems (Hontzsch, Bondby, Denmark) swimming flume (L × W × H, 40 × 10 × 10 cm swim chamber) at 28°C (200 W submersible heater, Hydor THEO, Bassano del Grappa, Vicenza, Italy). Fish were introduced to the swim chamber with low flow velocity, acclimated for 15 min, and then velocity was increased by 4 cms^−1^ at 5‐min intervals until fish were unable to maintain station (Plaut 2000; Conradsen and McGuigan 2015), and *U*_crit_ calculated following Brett (1964). WIK zebrafish are highly sexually dimorphic in swimming speed (Conradsen and McGuigan 2015; Conradsen et al. 2016; McGuigan and Aw 2017), and therefore males and females were assayed separately, with selection applied independently on both sexes. Fish were swum in groups; individual *U*_crit_ is highly repeatable when fish are swum in different groups (Conradsen et al. 2016), indicating that the assay captures information on individual performance capability.

A *U*_crit_ trial consisted of same‐sex siblings; after trial completion, each fish was allocated to one of three groups based on their swimming performance relative to their siblings within that trial: fast (F), slow (S), or not selected. For each replicate family, 18–33 (average 26; 15 males and 12 females) fish were assayed over two to four trials. For the families that successfully produced offspring in each selection treatment (see below for further details), selection was applied more strongly on the more numerous sex (males, ∼68% of males versus ∼84% of females allocated to a selection treatment). In males, fish allocated to a selection treatment had an average *U*_crit_ 1.2 SD above (F) or 1.1 SD below (S) their family mean swimming speed, with an average of 34 cms^−1^ difference in speed between the treatment means. In females, average *U*_crit_ was 0.9 SD above (F) and 0.8 SD below (S) the replicate family mean, and the average speed difference between treatments was 18 cms^−1^. A total of six to 12 fish were allocated to each selection treatment within each ENU family, with, at most, one more male than female within each group.

Following allocation to a treatment, fish were bred via full‐sibling mating with other individuals in the same ENU family and selection treatment group. Using crosses within independent ENU treatment derived families allowed us to generate a population where individuals carried zero, one or two copies, at expected proportions of 0.25:0.50:0.25, respectively, of each mutation induced in their grandsire (Schneeberger 2014). Fish were bred both in small (two to three fish per sex) groups, and using in vitro fertilization, which ensured relatively equal contributions from all selected parents. Each replicate within each speed selection treatment was reared in three or four tanks to ensure that common environment effects could be partitioned from evolved differences between treatments. Fish were reared under the same standard conditions as described above.

### EFFECTS OF SELECTION ON SWIMMING SPEED AND REPRODUCTIVE FITNESS

We determined the effect of the selection on the swimming speed and reproductive output of the subsequent, F2, generation (i.e., grandchildren of mutagenized males). *U*_crit_ was assayed as described above, with each swimming trial consisting of six same‐sex fish from one replicate tank within a treatment and family. A total of 390 fish were swum in 65 swimming trials. Following their swimming trial, fish were photographed, and size (standard length) determined as described in Conradsen et al. (2016).

Reproductive success was assessed through crosses both within and among speed treatments and ENU families, with the goal of obtaining equal numbers of clutches across all possible combinations of family and treatment. After initial observation that individual breeding pairs rarely produced clutches, crosses were set up with one to three females and two or three males. All individuals of one sex were from the same family and treatment, ensuring that any embryos were the result of crosses between the intended groups. More than 300 breeding attempts were made over a 66‐day period (fish were 229–317 dpf). Low breeding success is likely indicative of poor fitness rather than simple husbandry issues as outbred populations of the same age maintained under the same conditions had high breeding success. We exclude from the analyses any clutches with 0% viability (13% of all clutches, including 5% slow by slow, 2% fast by fast, and 6% slow by fast crosses) to avoid any problem from zero inflation. A total of 58 clutches with >0% viability were available for analysis (Table S1). By focusing only on those matings that produced some viable embryos, we likely underestimate fitness effects of mutation load.

Eggs were collected, washed in embryo media (saline with 10^−5^% methylene blue), counted, and up to 50 eggs placed in a petri dish (90 mm × 15 mm) with embryo media; a maximum of three dishes of 50 eggs were retained per clutch. Embryos were reared in a controlled environment cabinet (Laboratory Equipment Pty Ltd model PGX‐450B) at 28°C, 12‐h:12‐h light:dark cycle. Due to logistics, viability was recorded on 3, 4, or 5 dpf. There was no difference among cross type (SS, FF, SF/FS) in when viability was recorded. The majority of deaths occurred early, and viability at 3 dpf was strongly indicative of viability at 5 dpf. Although it is possible to determine whether cell division was initiated (Kimmel et al. 1995), for eggs where zygote development did not initiate we could not determine whether males did not release sperm, released sperm was inviable or eggs were inviable. Moreover, it was logistically impractical to determine whether embryogenesis initiated in all eggs, particularly when multiple mating groups were successful on a single day and several hundred to thousands of eggs needed to be sorted. We, therefore, report a measure of viability that confounds the parental traits of sperm release and gamete viability with the offspring trait of zygote viability.

### DATA ANALYSES

All analyses were conducted using PROC GLIMMIX in SAS (version 9.4; SAS Institute Inc., Cary, NC, USA).

#### Swimming speed

Fish within a swimming trial all came from the same selection treatment, the same replicate family, and the same rearing tank (nested within treatment and family), and might be considered pseudo‐replicates. We therefore conservatively treated replicate rearing tanks (three or four per family and treatment) as the smallest experimental unit for determining the evolved response to selection on swimming performance. Qualitatively the same results were obtained, and the same conclusions drawn, when individual fish records were analyzed.

We determined the effect of selection treatment, sex, and age on tank‐mean swimming speed through mixed model analysis of covariance (ANCOVA), fit using maximum likelihood. Selection treatment and sex were fit as categorical fixed effects, and age as a continuous covariate. We initially fit all possible interactions among the three fixed effects, and re‐fit the model excluding all interactions with age that were not significant to avoid biasing estimates of main effects (Engqvist 2005). Selection treatment by replicate ENU family interaction and replicate ENU family were fit as random effects, along with the residual (among replicate rearing tanks within family and treatment). There was no significant heterogeneity among families in their response to the selection treatment (Supporting Information).

Although eight replicate F1 families were subjected to divergent selection, three families failed to produce any surviving offspring. We analyzed *U*_crit_ of F1 parental fish to test our overall hypothesis that slower swimming fish had lower reproductive fitness than faster swimming fish. We analyzed the average *U*_crit_ of each swimming trial per family, and used maximum likelihood to fit a mixed model ANCOVA in which offspring viability (0 or 1) and sex were fixed effects, age a continuous covariate, and ENU family a random effect. Again, all interactions among fixed effects were initially fit and the analysis was re‐run excluding nonsignificant covariate terms.

#### Reproductive success

In the analysis of reproductive success, the response trait (viability) was measured in crosses among ENU families and selection treatments. This breeding design is analogous to a partial diallel across environments, although we cannot interpret effects as estimates of additive or nonadditive (dominance) genetic variance as we could if our ENU families corresponded to genetically homogeneous inbred lines (Lynch and Walsh 1998). Nonetheless, the diallel breeding design provides a useful framework for the analysis of our data. Where the proportion of viable embryos per replicate Petri dish was the response variable, we used maximum likelihood to fit a mixed model ANCOVA to determine whether treatment, which had three categories (slow by slow, fast by fast and slow by fast), parental age (average age of both parents, a continuous covariate), inbreeding (categorical variable indicating if crosses were within or among families) or the interaction between these effects contributed to variation in reproductive success. Again, we removed non‐significant covariate interaction terms to obtain the final results. Five random effects were modeled, accounting for the general effect (GCA) of the replicate ENU families, the effect of the specific combination of the ENU families crossed (SCA), the effect of the interaction between selection treatment and the GCA, the effect of the interaction between selection treatment and the SCA, and the effect of replicate clutches of the same cross (treatment and ENU family combination). The residual was the variance among replicate Petri dishes per clutch. To fit the random effects, we adapted the SAS IML code from Isik (2009) to reduce the complexity of our design by pooling reciprocal crosses, treating crosses where an ENU family within a treatment supplied the mother as equivalent to crosses where that family and treatment supplied the father. Although sex‐specific genetic effects on viability might occur, our primary focus here was on whether the selection treatment affected mean reproductive success. Analysis of random effects again showed no significant heterogeneity among families in their response to the selection treatment (Supporting Information).

## Results and Discussion

### DIRECT RESPONSE TO SELECTION ON SWIMMING SPEED AND IMPLICATIONS FOR SELECTION ON MUTATION LOAD

**Figure 1:**
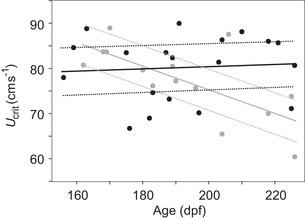
The relationship between critical swimming speed (*U*_crit_, cms^−1^) and age (days postfertilization [dpf]) for offspring of slow (gray circles and lines) versus fast (black circles and lines) swimming parents. The least‐squares means for each tank are plotted, with the least‐squares regression lines shown for each selection treatment (solid lines); dashed lines indicate least squares regression slopes for ±1 standard error on least‐squares mean estimates.

Our a priori expectation was that swimming speed would negatively correlate with mutation load due to the condition‐dependent nature of whole‐organism performances (Husak and Lailvaux 2017; Lailvaux and Husak 2017). F2 offspring of the F1 fish assigned to fast versus slow swimming speed treatments showed significant heterogeneity in the relationship between age and swimming speed (*F*_1,60_ = 7.27, *P* = 0.0091; Fig. 1; Table S2). On average, offspring of slow swimming parents declined in *U*_crit_ by 0.284 ± 0.105 cms^−1^ per day, while speed was independent of age in the fast treatment (0.056 ± 0.070 cms^−1^ per day) (Fig. 1). The relationship between swimming speed and age was not mediated through age‐dependent size: size did not vary with age (*F*_1,61_ = 1.02, *P* = 0.3168; Table S2) and *U*_crit_ did not vary with size (size by treatment: *F*_1,58_ = 0.39, *P* = 0.5325; main effect of size: *F*_1,8_ = 0.10, *P* = 0.7616; Table S2).

Divergence in age‐dependent swimming speed mimics the pattern observed between populations of WIK zebrafish known to differ in mutation load (McGuigan and Aw 2017). High mutation load zebrafish declined in speed with age, as observed here for the slow swimming selection treatment, while the paired wild‐type (low mutation load) population exhibited age‐independent speed, as observed here for the fast swimming selection treatment. Thus, our population of F2 offspring of slow swimming parents behaved as expected for a population of individuals carrying relatively many mutations, whereas their cousins, offspring of fast swimming F1 fish, behaved as expected for a population carrying fewer mutations.

**Figure 2:**
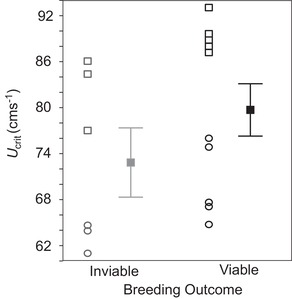
Reproductive success relative to parental swimming speed (*U*_crit_) in F1 fish. Mean *U*_crit_ (cms^−1^) for the three families that failed to produce F2 offspring (inviable) versus the five families that bred successfully (viable). For each group, family means are plotted for males (squares) and females (circles), with the grand mean (±1SE) (across all families and both sexes) in each group plotted to the right. Note: within each sex, there is no overlap in speed between viable and inviable family means.

Further evidence that deleterious mutation load reduces locomotor performance comes from the observation that F1 families that were unable to reproduce swam slower than reproductively successful F1 families. The three ENU F1 families with no F2 offspring swam, on average, 5.92 ± 3.13 cms^−1^ slower than fish from the five F1 families with viable F2 offspring, a significant decrease in swimming speed (*F*_1,6_ = 7.92, *P* = 0.0306; Fig. 2; Table S3). There was no effect of age on speed in these F1 fish (age: *F*_1,10_ = 1.33, *P* = 0.2762; age by F2 survival: *F*_1,10_ = 0.19, *P* = 0.6686; Table S3), which were all more similar in age when their *U*_crit_ was assayed than were fish in the F2 cohort, reflecting the time taken to assay the greater number of fish in the F2 generation.

### INDIRECT RESPONSE OF REPRODUCTIVE SUCCESS AND IMPLICATIONS FOR SELECTION ON MUTATION LOAD

**Figure 3:**
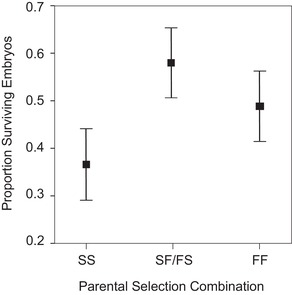
Reproductive success of crosses within and among swimming speed selection treatments. Clutch mean viability (average proportion of surviving embryos, least‐mean squares ±1 SE) is plotted for crosses between F2 fish derived from the slow swimming selection treatment (SS), the fast and slow swimming selection treatments (SF/FS) and the fast swimming selection treatment.

The three types of F2 crosses, among slow, among fast, or between fast and slow, differed significantly in viability (*F*_2,8_ = 5.58, *P* = 0.0305; Table S4). Specifically, as expected if slow swimming fish carry a greater mutation load, crosses among fish in the slow swimming selection treatment had significantly lower viability (least squares mean ± SE: 0.371 ± 0.078) than crosses either within the fast swimming treatment (0.495 ± 0.078) or between the fast and slow swimming treatments (0.577 ± 0.076) (planned contrast of slow to both fast and fast by slow: *F*_1,8_ = 9.38, *P* = 0.0155; Fig. 3). Reproductive success decreased significantly with parental age, declining at 0.0030 (± 0.0008) embryos surviving per increase of one day of average parental age (*F*_1,97_ = 14.50, *P* = 0.0002), but there was no evidence that reproductive success declined with age at different rates for the three types of crosses (*F*_2,95_ = 0.95, *P* = 0.3911).

Although we were unable to directly determine the causes of low reproductive success, embryo death was strongly implicated. If fish in the slow swimming selection treatment had low gamete viability, we would expect that reproductive success would be low for any cross these fish were involved in. However, crosses of slow to fast swimming selection treatment fish had relatively high mean viability (Fig. 3). Nonetheless, we cannot rule out that fish in the slow swimming selection treatment adjusted which gametes they released depending on the selection treatment of the potential mates they encountered.

Crosses were made both within and among families derived from each ENU‐treated grandsire. If recessive ENU‐induced mutations contributed to reproductive success, crosses within families, where offspring have a 25% probability of inheriting two copies of the same ENU‐induced mutation, should have reduced offspring survivial compared to among‐familiy crosses. Further, if, as expected, the slow swimming selection treatment carries more ENU‐induced mutations than the fast swimming selection treatment, within family crosses within the slow swimming selection treatment should have the lowest viability. In contrast to these predictions, there was no evidence that crosses within family had lower reproductive success that crosses among families (inbreeding: *F*_1,97_ = 0.09, *P* = 0.7657; Table S4) or that treatments differed in the effect of inbreeding on reproductive success (inbreeding by treatment: *F*_1,97_ = 1.91, *P* = 0.1698; Table S4). Our data are, therefore, not consistent with fully recessive alleles as the major cause of reduced reproductive success.

Mutation load can be purged with reduced effects on population growth rate if mutations interact epistatically, although there is little empirical evidence for this (Agrawal and Whitlock 2012; Jasmin and Lenormand 2016). We suggest that our results are consistent with synergistic epistasis where selective death occurs in individuals with a mutation load above a threshold, or that selective death increases with mutation load. Such a mechanism has been supported in the green algae *Chlamydomonas reinhardtii*, where competitive fitness negatively correlated with total mutation number (Kraemer et al. 2017). If, as we have suggested, F2 offspring of slow swimming F1 fish carry relatively many mutations, crosses within the slow swimming treatment will result in a high average mutation number, irrespective of whether crosses are made within or among families. In contrast, crosses to fish from the putatively unloaded fast swimming selection treatment will result in a lower average number of mutations passed to embryos. This mechanism is consistent with our observations of no effect of inbreeding (within versus among ENU family crosses), and the difference in reproductive success of crosses within the slow swimming selection treatment versus between selection treatments.

## Conclusions

Mutations frequently arise in populations of eukaryotes and are eventually removed by selection (Eyre‐Walker and Keightley 2007; Halligan and Keightley 2009). However, we have relatively limited understanding of the mechanisms of selection against mutation load (Agrawal and Whitlock 2012). Here, we show that mutagenesis provides a powerful approach for investigating selection against mutation load. Although the loads induced are not biologically realistic, by increasing the frequency of deleterious mutations, such experiments allow us to visualize how load might vary with phenotype and fitness in experiments with manageable sample sizes. Our results provide evidence of concordant selection against mutation load via whole‐animal performance and reproductive success.

If the observed low reproductive success of putatively high mutation load fish reflects embryo inviability, it has important implications for selection against mutation load. Selective elimination of mutations early in the life cycle is predicted to mitigate effects of selective death on population growth (Agrawal and Whitlock 2012) and has implications for detecting selection (Hadfield 2008). Although many taxa are known to have high mortality early in life (Levitis 2011), the role of mutation load in determining this mortality is not well understood (Plough et al. 2016). Similarly, recent work suggests selection acting on gametic variation within individuals might be an underappreciated force in evolution (Immler and Otto 2018). Populations that putatively differ in mutation load can differ in fitness components early in life, but not as adults (Kolb and Durka 2013). These observations suggest that our understanding of selection on mutation load might be advanced through further studies of selection acting directly on gametes and embryos.

Our experimental design took advantage of Mendelian segregation within families when applying divergent selection on siblings. Recent theoretical work has highlighted the high variance in per locus relatedness among siblings generated by Mendelian segregation (Hill and Weir 2011). Considered within the context of viability selection on gametes or zygotes, mutation load might be very different between offspring that survive versus those that do not, but this variation might not manifest as large, statistically detectable, differences in reproductive fitness among parents in well‐adapted populations. Suggestively, significant differences in fitness within full‐sibling families have been reported (e.g., McGuigan and Blows 2009; Sztepanacz and Rundle 2012; Plough et al. 2016), and within‐family selection can limit fitness declines due to accumulation of mutation load (McGuigan et al. 2011b). However, further work is required to determine the effect of selection on mutation load at different scales.

Together, the reduced reproductive success of crosses among offspring of slow swimming F1 fish and the relatively slow swimming speed of F1 families that were unable to reproduce provide strong evidence that locomotor performance is mutationally correlated with reproductive success. Our results, therefore, contribute support for the expectation that performance is genetically correlated with fitness within populations (Nicoletto 1995; Irschick et al. 2008; but see Lailvaux et al. 2010). However, our results also suggest challenges to detecting this effect in wild populations where exact information on, for example, age and early‐life environmental experiences is lacking, and where individuals are unlikely to carry such high mutation loads.

## DATA ACCESSIBILITY

Data will be made publicly available upon acceptance.

Associate Editor: A. Charmantier

## Supporting information

Supporting Information

**Table S1**. The number of clutches obtained from crosses within and among swimming speed selection treatments (Treat) and replicate families per treatment.
**Table S2**. Results of maximum likelihood analyses of the effect of selection treatment, sex and age or size on critical swimming speed (*U*_crit_) or size (SL).
**Table S3**. Results of maximum likelihood analysis of swimming speed (*U*_crit_) in F1 fish that either successfully produced offspring (viable) or failed to produce offspring (inviable).
**Table S4**. Maximum likelihood analysis of reproductive success of crosses within and among F2 families testing for effects of cross type (Cross: within or among fast and slow selected treatments), age (average age of parents in days) and inbreeding (whether or not the cross was within a family) on proportion of embryos surviving.
