# Supplementary material for: Do slower movers have lower reproductive success and higher mutation load?
Source: Evol Lett. 2018 Nov 12;2(6):590–8. doi: 10.1002/evl3.87 (PMC6292707; doi:10.1002/evl3.87)
Supplement: Supplementary file 1 — Supporting Information [file EVL3-2-590-s001.pdf]

Supporting Information: Random Effect Results

In our mixed model analyses of the direct and indirect responses to divergent within family selection on swimming speed, the random effects capture information both on the heritable variation in the traits, and further information on the consistency of response to selection across the replicate ENU treatment families. Importantly, the significance of the fixed effect of selection treatment takes into account any among-family heterogeneity within each treatment. However, the extent of variation might also be of interest. As detailed below, there was no evidence that ENU families responded differently to the selection treatment, with the null hypothesis of zero variance for the genotype by environment interaction not rejected for either phenotype.

Genotype and Genotype by Environment Effects on Swimming Speed

We fit a restricted maximum likelihood model (implemented in PROC GLIMMIX in SAS v. 9.4; SAS, Institute Inc. Cary NC, USA) with treatment and age as fixed effects, with no interaction between them. We treated the data as a random regression, estimating the variation in how ENU families responded to the selection treatment (treatment by family interaction) and among ENU families for three parameters: swimming speed (intercept), the slope of swimming speed with age, and the intercept-slope covariance. As individual fish were only swum once, a single residual term was estimated. Both swimming speed and age were variance standardised (standard deviation =1, mean =0) to allow us to consider them on the same scale (Martin et al. 2011). We tested the null hypothesis that random effect variance estimates were zero by applying log-likelihood ratio tests comparing nested models in which the effect of interest was estimated with models where that effect was held to zero; this log-likelihood ratio test follows a chi-square distribution with degrees of freedom determined by the number of parameters tested, and a mixture reflecting the one-tailed test imposed by the fact that variances cannot be less than zero (Self and Liang 1987). Intercept-slope covariance was not supported for either random effect.

Overall, family by treatment accounted for 10.7% of observed variance in swimming speed among tanks of F2 fish. However, there was no statistical support for rejecting the null hypothesis of zero family by treatment variance for either intercept (8.0% of variance: X^2 = 0.50, P = 0.2394) or slope (2.7% of variance: X^2 = 1.63, P = 0.1007). Therefore, the observed evolutionary response in swimming speed appears consistent across replicates in which mutation was independently induced, and selection was independently applied.

Replicate ENU families did differ significantly in swimming speed, as expected give that each family inherited mutations independently induced in different founder males. Among family variance accounted for 33.9% of the variance in U_{crit}, with statistical support for among-family variation in mean swimming speed (intercept: X^2 = 3.00, P = 0.04165), but not in how fish changed speed with age (slope: X^2 = 0.00, P = 0.4993).

Genotype and Genotype by Environment Effects on Reproductive Success

For reproductive success, the model contained four random effects of interest, two genotype by environment effects and two genetic effects. Again, the independent replicates (ENU families) appeared to be similarly affected by the selection on swimming speed, with little variance in reproductive success associated with treatment-specific effects of the ENU family (i.e., treatment by GCA: GCA: 0%, X^2 = 0.00, *P* = 0.5000) or treatment-specific effects of the combination of ENU families crossed (i.e., treatment by SCA: 3.3%, X^2 = 0.10, *P* = 0.3764). Although statistical support was weak, ENU families appeared to differ in their reproductive success, with 18.4% of the variance attributed to a general effect of ENU family (X^2 = 2.34, *P* = 0.0630) and 12.1% to the specific combination of ENU families mated (X^2 = 1.51, *P* = 0.1092).

Literature Cited

Martin, J. G. A., D. H. Nussey, A. J. Wilson, and D. Reale. 2011. Measuring individual differences in reaction norms in field and experimental studies: a power analysis of random regression models. Methods in Ecology and Evolution 2:362-374.

Self, S. G. and K.-Y. Liang. 1987. Asymptotic properties of maximum likelihood estimators and likelihood ratio tests under nonstandard conditions. Journal of the American Statistical Association 82:605-610.
