# Supplementary material for: Do slower movers have lower reproductive success and higher mutation load?
Source: Evol Lett. 2018 Nov 12;2(6):590–8. doi: 10.1002/evl3.87 (PMC6292707; doi:10.1002/evl3.87)
Supplement: Supplementary file 2 — Table S1. The number of clutches obtained from crosses within and among swimming speed selection treatments (Treat) and replicate families per treatment. Table S2. Results of maximum likelihood analyses of the effect of selection treatment, sex and age or size on critical swimming speed (U crit) or size (SL). Table S3. Results of maximum likelihood analysis of swimming speed (U crit) in F1 fish that either successfully produced offspring (viable) or failed to produce offspring (inviable). Table S4. Maximum likelihood analysis of reproductive success of crosses within and among F2 families testing for effects of cross type (Cross: within or among fast and slow selected treatments), age (average age of parents in days) and inbreeding (whether or not the cross was within a family) on proportion of embryos surviving. [file EVL3-2-590-s002.docx]

**Table S1.** The number of clutches obtained from crosses within and among swimming speed selection treatments (Treat) and replicate families per treatment. Clutches for which no embryos survived are shown in brackets. “F” indicates fast-swimming parents were selected; “S” indicates that slow-swimming parents were selected. The replicate ENU treatment derived families are numbered 1 to 5. The female parents (mother) are shown in columns and male parents (fathers) in rows.

|  |  |  | | Female | | | | | | | | | |
| --- | --- | --- | --- | --- | --- | --- | --- | --- | --- | --- | --- | --- | --- |
| Family | | | | 1 | | 2 | | 3 | | 4 | | 5 | |
|  | Treat | | | F | S | F | S | F | S | F | S | F | S |
| Ma l e | 1 | | F | 1 | 1 |  |  |  | 1 | 1 |  |  |  |
|  |  |  | S | 1 | 1 | 1 | 1 | 1 | 1 | 1 | 1 |  |  |
|  | 2 | | F | 2 | 1(1) | 2 | 1 |  | 1 | (1) |  |  |  |
|  |  |  | S | 3 | 2 | 1 | 5(2) |  | 1 | 1(1) | 1(1) |  |  |
|  | 3 | | F | 2 | 1 | (1) | 2 | 1(1) | (1) | 1(1) |  |  |  |
|  |  |  | S | 1(1) | 1 |  | 1 |  | 1 | 2 | (1) |  |  |
|  | 4 | | F | 1 | 1(1) | (1) | 1 | (1) | 1(1) | 3(1) |  |  | 2 |
|  |  |  | S | 4 | 1(1) |  | (1) |  | (1) |  | (1) |  | (1) |
|  | 5 | | F | 1 | 2 |  |  |  |  | 1 |  | 3(1) | 3 |
|  |  |  | S | 4 | 1 |  |  |  |  | (1) | 1 |  | 2 |

**Table S2.** Results of maximum likelihood analyses of the effect of selection treatment, sex and age or size on critical swimming speed (*U*_crit_) or size (SL). Analyses were conducted on tank means. Results are shown for a model in which all main effects and interactions were fit (full model), and for a model in which non-significant covariate (age or sex) interactions were removed. Significant effects are indicated (**P*< 0.05; ** *P*< 0.01; ****P*< 0.001).

| Model | Response | Effect | DF | F-Value | Pr>F |  |
| --- | --- | --- | --- | --- | --- | --- |
| Full | *U*_crit_ | Age | 1,58 | 2.91 | 0.0932 |  |
|  |  | Sex | 1,58 | 3.5 | 0.0665 |  |
|  |  | Treatment | 1,8 | 5.64 | 0.0448 | * |
|  |  | Treat*Sex | 1,58 | 1.47 | 0.2302 |  |
|  |  | Age*Sex | 1,58 | 0.07 | 0.7989 |  |
|  |  | Age*Treat | 1,58 | 6.08 | 0.0166 | * |
|  |  | Age*Treat*Sex | 1,58 | 1.69 | 0.1984 |  |
|  |  |  |  |  |  |  |
| Reduced | *U*_crit_ | Age | 1,60 | 2.72 | 0.1045 |  |
|  |  | Sex | 1,60 | 318.24 | <.0001 | *** |
|  |  | Treatment | 1,8 | 6.69 | 0.0323 | * |
|  |  | Treat*Sex | 1,60 | 0.84 | 0.3620 |  |
|  |  | Age*Treat | 1,60 | 7.27 | 0.0091 | ** |
| Full | *U*_crit_ | size | 1,58 | 1.36 | 0.2489 |  |
|  |  | Sex | 1,58 | 0.43 | 0.5137 |  |
|  |  | Treat | 1,8 | 0.36 | 0.5625 |  |
|  |  | Treat*Sex | 1,58 | 0.42 | 0.5189 |  |
|  |  | size*Sex | 1,58 | 0.07 | 0.7916 |  |
|  |  | size*Treat | 1,58 | 0.39 | 0.5325 |  |
|  |  | size*Treat*Sex | 1,58 | 0.32 | 0.5753 |  |
|  |  |  |  |  |  |  |
| Reduced | *U*_crit_ | size | 1,61 | 1.47 | 0.2307 |  |
|  |  | Sex | 1,61 | 192.81 | <.0001 | *** |
|  |  | Treat | 1,8 | 0.92 | 0.3656 |  |
|  |  | Treat*Sex | 1,61 | 1.41 | 0.2403 |  |
| Full | Size (SL) | Age | 1,58 | 0.85 | 0.3617 |  |
|  |  | Sex | 1,58 | 0.08 | 0.7809 |  |
|  |  | Treatment | 1,8 | 0.07 | 0.7971 |  |
|  |  | Treat*Sex | 1,58 | 1.8 | 0.1849 |  |
|  |  | Age*Sex | 1,58 | 0.87 | 0.3551 |  |
|  |  | Age*Treat | 1,58 | 0.06 | 0.8059 |  |
|  |  | Age*Treat*Sex | 1,58 | 1.61 | 0.2096 |  |
|  |  |  |  |  |  |  |
| Reduced | Size (SL) | AGE | 1,61 | 1.02 | 0.3168 |  |
|  |  | Sex | 1,61 | 50.49 | <.0001 | *** |
|  |  | Treat | 1,8 | 0.1 | 0.7616 |  |
|  |  | Treat*Sex | 1,61 | 0.82 | 0.3688 |  |

**Table S3.** Results of maximum likelihood analysis of swimming speed (*U*_crit_) in F1 fish that either successfully produced offspring (viable) or failed to produce offspring (inviable). Results are shown for a model in which all main effects and interactions were fit (full model), and for a model in which non-significant covariate (age or sex) terms were removed.

| Model | Response | Effect | DF | F-Value | Pr>F |  |
| --- | --- | --- | --- | --- | --- | --- |
| Full | *U*_crit_ | Age | 1,10 | 1.33 | 0.2762 |  |
|  |  | Sex | 1,10 | 3.22 | 0.1031 |  |
|  |  | Viability | 1,6 | 0.08 | 0.7813 |  |
|  |  | Viab*Sex | 1,10 | 3.09 | 0.1090 |  |
|  |  | Age*Sex | 1,10 | 1.51 | 0.2466 |  |
|  |  | Age*Viab | 1,10 | 0.19 | 0.6686 |  |
|  |  | Age*Viab*Sex | 1,10 | 3.19 | 0.1046 |  |
|  |  |  |  |  |  |  |
| Reduced | *U*_crit_ | Sex | 1,14 | 82.18 | <.0001 | *** |
|  |  | Viability | 1,6 | 7.92 | 0.0306 | * |
|  |  | Viab*Sex | 1,14 | 0 | 0.9801 |  |

**Table S4**. Maximum likelihood analysis of reproductive success of crosses within and among F2 families testing for effects of cross type (Cross: within or among fast and slow selected treatments), age (average age of parents in days) and inbreeding (whether or not the cross was within a family) on proportion of embryos surviving. Results are shown for a model in which all main effects and interactions were fit (full model), and for a model in which non-significant covariate (age or inbreeding) terms were removed. Two reduced models are shown, as the marginally significant cross*inbred term was initially retained.

| Model | Response | Effect | DF | F-Value | Pr>F |  |
| --- | --- | --- | --- | --- | --- | --- |
| Full | Proportion of viable embryos | Age | 1,95 | 9.30 | 0.0030 | ** |
|  |  | Cross | 2,8 | 1.44 | 0.2932 |  |
|  |  | Inbred | 1,95 | 1.20 | 0.2757 |  |
|  |  | Cross*Inbred | 1,95 | 3.81 | 0.0538 |  |
|  |  | Age*Inbred | 1,95 | 1.13 | 0.2904 |  |
|  |  | Age*Cross | 2,95 | 0.95 | 0.3911 |  |
|  |  | Age*Cross*Inbred | 1,95 | 3.33 | 0.0713 |  |
|  |  |  |  |  |  |  |
| Reduced |  | Age | 1,97 | 15.04 | 0.0002 | *** |
|  |  | Cross | 2,8 | 6.16 | 0.0240 | * |
|  |  | Inbred | 1,97 | 0.09 | 0.7657 |  |
|  |  | Cross*Inbred | 1,97 | 1.91 | 0.1698 |  |
|  |  |  |  |  |  |  |
| Reduced |  | Age | 1,97 | 14.50 | 0.0002 | *** |
|  |  | Cross | 2,8 | 5.58 | 0.0305 | * |
|  |  | Inbred | 1,97 | 0.10 | 0.7484 |  |
